# Multiple induced seismicity mechanisms at Castor underground gas storage illustrate the need for thorough monitoring

**DOI:** 10.1038/s41467-022-30903-6

**Published:** 2022-06-17

**Authors:** Víctor Vilarrasa, Silvia De Simone, Jesus Carrera, Antonio Villaseñor

**Affiliations:** 1 Institute of Environmental Assessment and Water Research, Spanish National Research Council (IDAEA-CSIC), Barcelona, Spain; 2 Mediterranean Institute for Advanced Studies, Spanish National Research Council (IMEDEA-CSIC), Esporles, Spain; 3 Associated Unit: Hydrogeology Group (UPC-CSIC), Barcelona, Spain; 4 Univ Rennes, CNRS, Géosciences Rennes, UMR 6118, 35000 Rennes, France; 5 Institute of Marine Sciences, Spanish National Research Council (ICM-CSIC), Barcelona, Spain

**Keywords:** Seismology, Hydrogeology, Geophysics, Natural gas

**arising from** Cesca et al. *Nature Communications* 10.1038/s41467-021-24949-1 (2021)

A recent publication by Cesca et al.^1^ reanalyzes and expands seismic data to identify hypocenters of observed seismicity induced by the Castor Underground Gas Storage (UGS) operations. Their results confirm those of previous studies^2,3^ that earthquakes occurred below the storage formation on a fault dipping opposite from the Amposta fault, which bounds the reservoir. However, two important sets of disagreements require revising the conclusions by Cesca et al.^1^: the depth of hypocenters and the processes leading to seismicity. Inaccurate estimates of hypocenters location and partial consideration of the physical mechanisms that induce seismicity may imply endangering future deep underground projects.

Cesca et al.^1^ located the seismicity at 3 km depth, within sedimentary deposits. We consider this depth highly unlikely for two reasons. First, moderate to large earthquakes nucleate at larger depths, not in shallow formations^4,5^. Besides, moderate earthquakes rarely occur in sedimentary rocks where failure tends to be ductile because of their moderate strength. That is, the rock is crushed and deformation becomes plastic, i.e., aseismic. In agreement with these two considerations, the observed magnitude of induced earthquakes positively correlates both with depth and rock strength (see compilation of induced events in sedimentary and crystalline rock by Evans et al.^6^ and McGarr^7^). The magnitude of the largest induced earthquakes at Castor (M4.1) is far too high for a depth of 3 km in sedimentary rock as suggested by Cesca et al.^1^.

Beyond the above “qualitative” reasoning, our second set of reasons against a shallow seismicity at the Castor UGS refers to the adopted velocity model and the importance of the seismic velocities of the region to accurately determine focal depths. As also illustrated by Cesca et al.^1^ in their Fig. 4d, estimated depths can vary several kilometers depending on the adopted velocity model. Of all the analyzed velocity models, Cesca et al.^1^ have chosen the one that yields the shallowest earthquake locations. Similarly, Juanes et al.^8^ obtained a detailed 3D velocity model of the region but chose a simple 1D model that provided the shallowest locations, which allowed explaining the induced seismicity by pore pressure buildup. Instead, Villaseñor et al.^2^ used local seismic data and well log information to derive a velocity model for the region. Using this model and waveform inversion, they obtained focal depths that range from 4 to 10 km. Some of the uncertainty on focal depth results would have been reduced if an adequate monitoring network had been in place. However, the available network lacks stations near the injection site, with the closest station located more than 20 km away. Such a poor array has caused the current debate to be centered on the depth of induced earthquakes, rather than on determining their cause.

Understanding the seismicity triggering mechanisms is important to prevent similar accidents in the future (not to mention to recover the public confidence on scientists and engineers). Cesca et al.^1^ suggest that the seismicity at Castor was induced by pore pressure buildup. We disagree with this hypothesis for several reasons. First, the high permeability of the storage formation resulted in a low pore pressure buildup at the injection depth, not exceeding 0.8 MPa according to the company. Second, this buildup could not be transferred 1 km below. Cesca et al.^1^ assume a hydraulic diffusivity of 0.5 m^2^/s. This value is two to three orders of magnitude larger than what should be expected at the sedimentary formations beneath the Castor reservoir, where nearby wells show alternations of high- (sandstones and limestones) and low-permeability (shales and evaporites) rock layers below the depth of the storage formation^9^. Hydraulic connection between the storage formation and the depth of the earthquakes requires the existence of some high-permeability conduit or fault. While such faults may occur in fragile crystalline rocks under favorable stress conditions, it is unlikely that they display significant permeability in the soft rock below the site. For a more reasonable, albeit highly uncertain, diffusivity, *D*, of 10^−3^ m^2^/s, it would take some 30 year for a small pressure to reach 1 km below the storage formation (*t* = *r*^2^⁄*D*, where *t* is time and *r* is distance). Finally, the largest earthquakes occurred 20 days after an injection of 15 days. If diffusivity had been sufficiently large for a significant pressure buildup propagation at depth, it should have also caused overpressure to dissipate by the time of the largest earthquakes.

While pressure build-up is the traditional explanation for mechanical destabilization^10^, numerous other mechanisms can intervene (Fig. 1), including thermal stresses^11^, deformation-induced pressure changes^12^, and shear slip stress transfer^13^. Vilarrasa et al.^3^ suggested that the delayed seismicity at Castor UGS was driven by the combination of three mechanisms: buoyancy, stress transfer driven by aseismic slip of the Amposta fault, and fluid flow to regions destabilized by deformation but maintained stable by deformation-induced pressure drop. An important issue about the triggering mechanism, which ultimately motivates this comment, is that we strongly disagree with two of the statements made in Cesca et al.^1^ The authors state “Another reason for the low seismogenic potential of gas storage operations could be that gas injection schedules are typically designed and engineered not to exceed the stress conditions existing prior to or during the original reservoir production”. We contend that in extensional stress settings, like the Valencia Trough, the injection schedule or the stress computed with conventional models are not key factors. Instead, seismicity is activated by the vertical stress changes caused by buoyancy. Archimedes’ principle states that the buoyancy force equals volume multiplied by the density of the displaced fluid (actually density contrast, when concerned with changes in stress). Therefore, the key destabilizing factor in extensional settings is the volume of the storage facility, which is not accounted for in models such as the one by Cesca et al.^1^ and, we fear, in the design of most deep-injection operations. The need for full poroelastic models and for proper integration of monitoring data has been argued for long (e.g., Juanes et al.^14^). In this sense, the second statement “Using… methods… allows us to reconstruct… proving that this is possible despite the lack of a dense local network” is questionable at best. Comparing the relocation and focal mechanisms presented now with those calculated by Cesca et al.^15^, it is clear that it has taken a lot of time and effort to reach a conclusion we do not share. Worse, this conclusion might be construed to interpret that sophisticated inversion might overcome the limitations of an inadequate monitoring network. We contend that one of the lessons learnt from Castor is just the opposite.

**Fig. 1:**
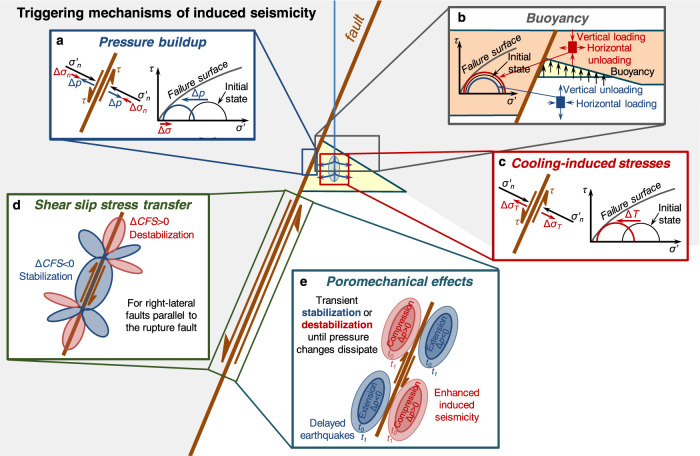
Potential triggering mechanisms of induced seismicity in geo-energy applications. **a** Pore pressure induced by fluid injection reduces the effective stress, approaching failure conditions; pore pressure changes induce compressive poroelastic stress that alters the initial stress state; **b** if the injected gas is buoyant, buoyancy causes a stress redistribution around the reservoir that may destabilize surrounding faults; **c** the injected fluid generally reaches the reservoir at a cooler temperature than the rock, leading to a cooling-induced stress reduction that brings the stress state closer to failure conditions; **d** seismic and aseismic slip induce a static stress transfer that stabilizes regions around the slipped area and destabilize others; **e** slip deforms the rock surrounding the slipped area, compressing and extending the rock ahead and behind the slip, respectively, which induces transient undrained pore pressure changes that tend to destabilize and stabilize, respectively, faults.

Physics-based modeling is a useful tool not only for understanding failures such as that at Castor UGS, but also for forecasting and eventually managing induced seismicity. The goal of doing it in real time can only be reached if a thorough monitoring system and decision-making protocol work properly. This is especially important at this stage in which several low-carbon geo-energy technologies compete for deep pore space: geothermal energy, geologic carbon storage, and subsurface hydrogen storage, all identified as essential to meet net-zero carbon emissions.

## Data Availability

The open access fully coupled thermo-hydro-mechanical code that we have used for our simulations can be downloaded at https://deca.upc.edu/en/projects/code_bright. The dataset used for our interpretation of the triggering mechanisms at Castor can be accessed at https://digital.csic.es/handle/10261/216863, with 10.20350/digitalCSIC/12553.
